# Effect of amino acids lysine and arginine on fracture healing in rabbits: A radiological and histomorphological analysis

**DOI:** 10.4103/0019-5413.55972

**Published:** 2009

**Authors:** Shivam Sinha, Satish Chandra Goel

**Affiliations:** Department of Orthopaedics, IPGMER, Kolkata, India; 1 Department of Orthopaedics, IMS, BHU, Varanasi, India

**Keywords:** Arginine, fracture healing, lysine, nitric oxide

## Abstract

**Background:**

Amino acids like arginine and lysine have been suggested to hasten the process of fracture healing by improving the local blood supply, supplementing growth factors, and improving collagen synthesis. We studied the role of lysine and arginine in the fracture repair process with regard to the rate of healing, probable mechanisms involved in the process, and mutual synergism between these agents.

**Materials and Methods:**

In an experimental study, 40 rabbits were subjected to ulnar osteotomy. They were distributed in control (14) and test groups (26). Twenty-six animals in the test group were fed with a diet rich in lysine and arginine. Both the groups were followed radiologically and histologically till union.

**Results:**

There was better healing of osteotomy in terms of better vascularization, callus formation, and mineralization in the test group. The time of healing in the test group was reduced by a period of 2 weeks.

**Conclusion:**

We conclude that amino acids like arginine and lysine may hasten fracture healing.

## INTRODUCTION

Fracture healing is a complex process. Many unknown factors come into act, even when reduction and/or fixation is stable but fractures fail to unite. Blaming it on poor bone quality, implant selection or poor patient compliance is not always justified. Poor biological healing can be overcome by the supplementation of certain factors which can promote fracture healing.^1^

The development of effective, safe, and cost-effective agents which could hasten fracture healing may open new avenues in the treatment of fractures. Worldwide research is going on regarding roles of certain growth factors and cytokines like IGF-I, BMP,^2^ TGF-β, and PDGF, which play important role in fracture healing.^3^

Amino acids like lysine and arginine have been reported to stimulate fracture healing in animal experimental models.^4^,^5^ Apart from their simple oral mode of administration, these amino acids provide a nontoxic and inexpensive option in this challenging field.

We studied the role of lysine and arginine in the fracture repair process with regard to the rate of healing, probable mechanisms involved in the process, and mutual synergism between these agents.

## MATERIALS AND METHODS

White New Zealand healthy adult rabbits (n = 40) irrespective of sex, weighing approx. 1.5 kg or more were utilized in the study. The work was carried out in Experimental Medicine and Surgery Research Laboratory at our institute. The study was protected under Cruelty for Animals Act (1960) and approved by institute's ethical committee.

### Experimental design

All the rabbits were fed with standard animal food. An oral formulation of lysine and arginine (Convina Research Lab, New Delhi) was administered to the animals in the test group through a dropper so that in addition to standard animal food, they received lysine at the rate of 47 mg/kg body weight (body wt) and arginine at the rate of 50 mg/kg body wt. Rabbits were anesthetized by a dose of 20 mg/kg of ketamine + 0.4 mg/kg of midazolam given intramuscularly.

Animals were placed in a lateral position on the operating table. The length of ulna was measured from the tip of olecranon to the wrist. The midshaft of ulna was exposed through an incision over ulnar aspect of the forearm. Transverse osteotomy of the midshaft of ulna was created using a bone saw. Local antibiotics (chloramphenicol) and betadine were instilled before closing the wound with interrupted 3-0 vicryl. Postoperatively, intramuscular antibiotics were administered. Test rabbits (26) were given a 50 ml solution containing 0.94 mg/ml of lysine and 1 mg/ml of arginine from postoperative day 1, by a dropper. The control group (14) was given only a normal rabbit diet.

Two animals each in the control group were sacrificed at 1, 2, 3, 4, 8, 12, and 20 weeks while in the test group two animals each were sacrificed at 1, 2, and 3 weeks and five each at 4, 8, 12, and 20 weeks.

### Outcome

#### Radiological evaluation

Rabbits were subjected to radiological assessment weekly starting from the immediate postoperative period till their proposed day of sacrifice. Radiological evaluation was carried out by the assessment of standard X-ray, assigning a score (XRS) based on the following criteria:

- Fracture gap
  Present—0, reduced—1, filled—2
- Bony bridge formation (visible on X-ray)
  Absent—0, irregular bone formation—1, canalization—2
- Amount of callus formation
  None—0, florid (periosteal)—1, endosteal (cortical healed)—2
- Appearance of fracture ends
  Sharp and fresh—0, irregular and healing—1, united—2, sclerosed and nonunion—1.
  Thus, a maximum score (XRS) that can be obtained is 8 and a minimum is 1.
  Final grading (X-ray grade = XRG) of the fracture on X-ray can be graded as

**Table:** 

X-ray grade, XRG	Score range, XRS	Healing status
0	1 to +2	No healing
1	+3 to +5	Partial healing
2	+6 to +8	Healed

#### Gross examination and manual palpation

Rabbits were killed by giving an overdose of ketamine and succinyl choline intramuscularly. Specimen of ulna was obtained and gross findings were observed like amount of callus, fragility, consistency, status of bony union and any abnormal mobility, and moldability of newly formed bone. The manual palpation score was awarded as per following findings: 0 = ununited; 1 = partially united, breaks on twisting; 2 = united, breaks on loading; and 3 = strong bony union.

#### Histological examination

Specimens cut for histological examination were decalcified and studied for fracture union. They were finally stained with hematoxyline and eosine. Fracture callus was assessed for degree of cellularity, amount of callus, cartilage, bone matrix formation, woven bone and mature bone formation, and medullary repair; cortical repair was scored as 1 = average (up to 25%), 2 = good (25–50%), 3 = very good (50–90%), and 4 = excellent (90–100%).

Thereafter a histopathologic grade (HPG) was awarded as follows:

**Table:** 

Grade 4:	Complete bone union
Grade 3:	Less than complete bony union as evidenced by the presence of a small amount of cartilage in fracture callus
Grade 2:	Complete cartilage union
Grade 1:	Incomplete cartilaginous union (evidenced by the retention of fibrous elements in the plate)
Grade 0:	Pseudoarthrosis formation or nonunion seen as an inconvertible cavity within the cartilage plate between fracture fragments containing blood or other fluid.

These observations were carried out by three separate observers to reduce the interobserver variation and each of the observers was blinded about the specimen's group to obtain an unbiased opinion.

### Statistical tools

The numerical parameters between the groups were assessed with the Mann–Whitney U-test while the various grades were compared with Fisher's exact test or chi-square test whichever was applicable. Statistical significance was set up at *P* < 0.05.

## RESULTS

In early stages of follow-up, i.e., at 1, 2, and 3 weeks, X-ray grading based on the proposed scoring system did not show any difference with overall XRG being zero. However, of the 22 test and 10 control rabbits (total animals = 32) who were subjected X-ray assessment at 3 weeks, 20 rabbits in the test group and 4 in the control group showed partial healing which corresponds to XRG = 1. These data were analyzed by Fischer's exact two-tailed test and the *P* value was found to be 0.005 which is highly significant (*P*<0.05). Test rabbits on gross morphological assessment showed a florid amount of callus which was fibrous but organizing; however, the fracture gap was visible and callus was easily breakable. In contrast, the control rabbits showed a lower amount of callus (on measurement with calipers) which was soft and fragile with abnormal mobility. Histological assessment of the two groups at 3 weeks revealed that [Table 1] though most of the parameters were similarly rated, there was gross difference in vascularity, Haversian system formation, and cortical and medullary repair between the two groups, with HPG being better in the test group.

**Table 1:** Histopathological grade and criteria for the assessment of histological parameters

Animal ID	Sacrifice (weeks)	Histological assessment								HPG
			
		Callus amount	Cells	Vascular	Cartilage/NBF	Haversian system	Bone matrix	Med. rep.	Cortic rep.	
T39	1	2	3	1	1	0	1	0	0	1
T38	1	2	3	1	1	0	0	0	0	0
T40	2	2	3	3	3	0	0	0	0	0
T41	2	2	2	3	3	0	1	0	0	1
T34	3	3	3	3	2	1	2	2	1	2
T35	3	2	3	1	2	3	2	3	3	3
T31	4	3	3	2	2	2	1	0	1	2
T30	4	3	3	2	2	1	2	2	1	2
T12	4	2	3	2	0	2	2	2	2	3
T13	4	2	3	2	3	2	3	2	2	3
T6	4	2	3	2	2	1	1	2	2	2
T16	8	2	3	1	0	2	2	1	2	3
T17	8	2	2	2	2	2	2	1	2	2.5
T3	8	3	2	3	3	3	3	3	3	4
T2	8	3	2	3	4	4	3	2	4	3
T18	8	3	3	2	2	2	3	2	3	3
T19	12	2	3	3	1	3	3	3	3	4
T14	12	2	2	3	1	3	3	3	2	4
T15	12	2	2	2	1	3	3	2	2	4
T24	12	3	3	3	1	4	4	4	2	4
T25	12	2	3	2	2	2	2	1	2	4
T8	20	2	2	3	3	3	3	3	2	3
T9	20	3	2	2	2	2	3	2	2	4
T1	20	3	3	4	3	3	3	2	3	4
T10	20	3	3	2	2	2	3	2	2	4
T11	20	3	3	3	2	3	3	2	2	4
C46	1	2	3	1	1	0	0	0	0	0
C47	1	2	3	1	1	0	1	0	0	1
C45	2	2	3	2	2	0	1	0	0	1
C44	2	2	3	2	1	0	1	0	0	1
C42	3	2	2	1	2	1	1	2	1	2
C43	3	3	2	2	2	0	1	1	0	1
C33	4	3	3	2	2	1	2	3	2	2
C32	4	3	2	2	2	1	1	2	2	2
C28	8	2	2	1	2	2	2	2	1	3
C29	8	3	2	1	2	3	2	2	2	3
C22	12	2	2	2	1	0	2	1	1	1
C23	12	3	2	2	2	2	3	2	2	3
C20	20	3	3	3	2	3	3	2	2	4
C21	20	3	2	2	2	2	2	3	2	3.5
*P* value	–	0.69	0.1	0.0311	0.44	0.0250	0.0426	0.39	0.0346	0.0371

T = test, C = control, HPG = Histopathological grade

**Figure 1:**
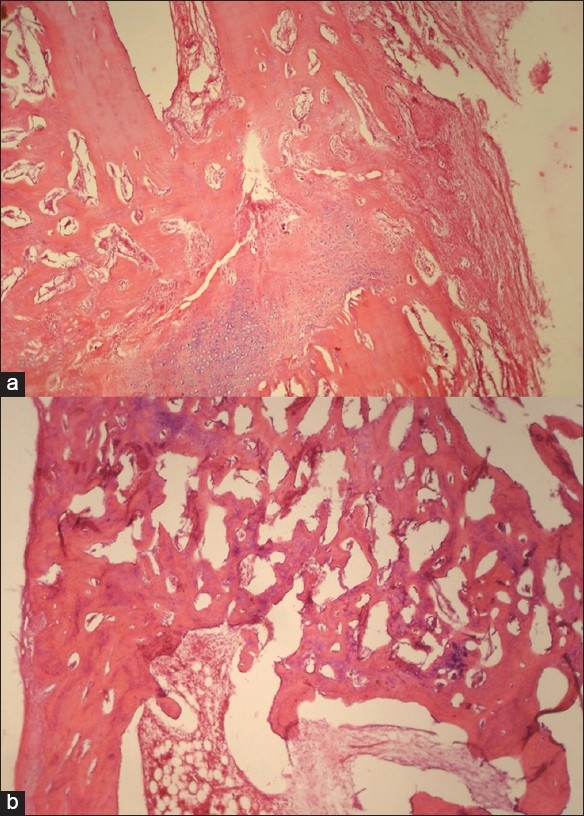
Comparison between histology of control and test at 12 weeks, showing partial union in control with an island of cartilage (a) and complete union in the test with marrow formation Haversian system development (b)

At 4 weeks, gross examination of the autopsy specimen showed bridging of the fracture gap by osseocartilagenous callus which is firm and does not break easily, but was deformable in a total of seven rabbits of both the groups who were sacrificed. Histology shows similar finding in both the groups, showing that healing in the control group which is going on a physiological rate in the control rabbits has kept pace with augmented healing in the test group. HPG did not show any difference in the two groups and points to a near-complete cartilaginous union (HPG = 2) [Figure 1]. X-ray evaluation of 20 test and 8 control animals (total animals = 28) at this period showed that all except one of the control rabbits had achieved a partial healing (XRG = 1); Fisher's exact test did not find this difference statistically significant (*P* = 0.286).

**Figure 2:**
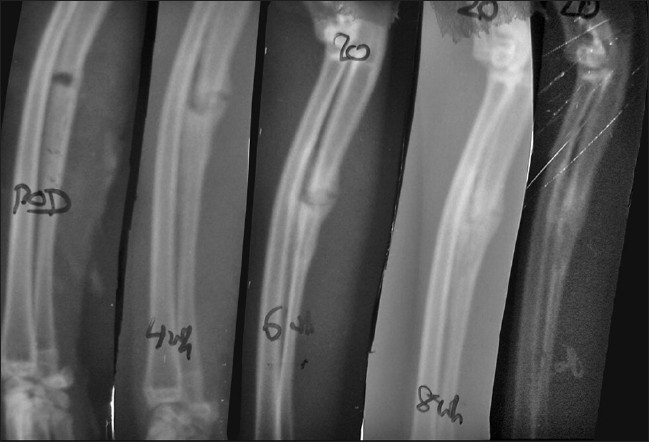
Sequential X-rays at 0, 4, 6, 8 and 12 weeks in control animal shows radiological union at 8 weeks

**Figure 3:**
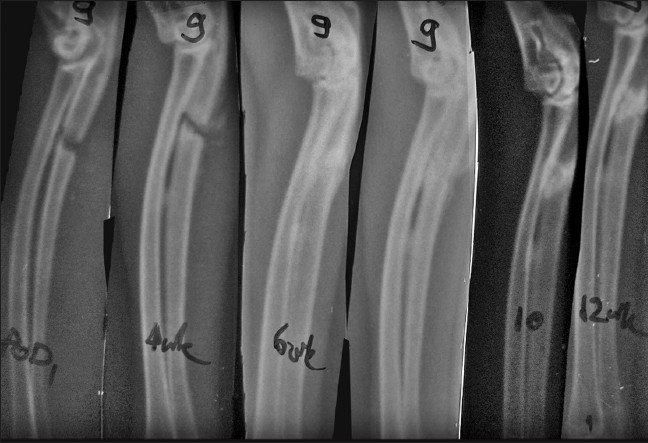
Sequential X-rays at 0, 4, 6, 8, 10 and 12 weeks in test animal shows radiological union at 6 weeks

At 8 weeks, gross autopsy revealed a bony union in both the groups (in all seven rabbits sacrificed at this period) but a higher amount of callus persisting in the control group. The assessment of histology reveals better remodeling in the test group [Table 1]. Vascularity at the fracture site had reduced in both the groups, with the overall HPG being either 3 or 4. X-ray scoring of the 21 rabbits (15 in test and 6 in control) ranges from a score of +5 to +7, thus, showing a transition from partial healing (×RG = 1) to complete healing (×RG = 2) [Figures 2 and 3]. However, one of the rabbits in the control group showed poor healing (×RS = −1, ×RG = 0). Data were analyzed by the chi-square test and the *P* value was found to be 0.125 which was not significant.

However, a significant difference was obtained when remaining 14 rabbits (10 test, 4 controls) were followed till 10 weeks with XRS [Table 2]. All test rabbits except one showed healing with XRG = 2, while one except all control rabbits showed such finding. Analysis with the chi-square test revealed a *P* value of 0.008, which is statistically very significant. This implies that in a period of 10 weeks postoperatively, there was difference evident radiologically between those supplemented with lysine and arginine, indicating that these components enhance the healing in the later part of bone remodeling, canal restoration, and medullary as well as cortical continuity and repair. Unfortunately, none of the rabbits were sacrificed at 10 weeks postoperatively as it was not included in the protocol.

**Table 2:** X-ray grades of rabbits at different time intervals and their analysis with tests of significance

Animal ID	Sacrifice (weeks)	X-ray evaluation		XRG	XRG	XRG	XRG	XRG	XRG	XRG
									
		1 week	2 weeks	3 weeks	4 weeks	6 weeks	8 weeks*	10 weeks*	12 weeks*	20 weeks
T39	1	0	–	–	–	–	–	–	–	–
T38	1	0	–	–	–	–	–	–	–	–
T40	2	0	0	–	–	–	–	–	–	–
T41	2	0	0	–	–	–	–	–	–	–
T34	3	0	0	1	–	–	–	–	–	–
T35	3	0	0	1	–	–	–	–	–	–
T31	4	0	0	1	1	–	–	–	–	–
T30	4	0	0	1	1	–	–	–	–	–
T12	4	0	0	1	1	–	–	–	–	–
T13	4	0	0	1	1	–	–	–	–	–
T6	4	0	0	1	1	–	–	–	–	–
T16	8	0	0	1	1	1	1	–	–	–
T17	8	0	0	0	1	1	1	–	–	–
T3	8	0	0	1	1	1	2	–	–	–
T2	8	0	0	1	1	1	1	–	–	–
T18	8	0	0	1	1	1	1	–	–	–
T19	12	0	0	1	1	1	1	2	2	–
T14	12	0	0	1	1	1	1	2	2	–
T15	12	0	0	1	1	1	1	2	2	–
T24	12	0	0	1	1	1	1	2	2	–
T25	12	0	0	1	1	1	1	2	2	–
T8	20	0	0	1	1	1	1	2	2	2
T9	20	0	0	1	1	1	1	2	2	2
T1	20	0	0	1	1	1	1	2	2	2
T10	20	0	0	1	1	1	2	2	2	2
T11	20	0	0	0	1	1	1	2	2	2
C46	1	0	–	–	–	–	–	–	–	–
C47	1	0	–	–	–	–	–	–	–	–
C45	2	0	0	–	–	–	–	–	–	–
C44	2	0	0	–	–	–	–	–	–	–
C42	3	0	0	1	–	–	–	–	–	–
C43	3	0	0	0	–	–	–	–	–	–
C33	4	0	0	0	1	–	–	–	–	–
C32	4	0	0	1	1	–	–	–	–	–
C28	8	0	0	1	1	1	2	–	–	–
C29	8	0	0	1	1	1	2	–	–	–
C22	12	0	0	0	0	0	0	0	0	–
C23	12	0	0	0	1	1	1	2	2	–
C20	20	0	0	0	1	1	1	1	2	2
C21	20	0	0	0	1	1	1	1	1	2
*P* value	–	0	0	0.005	0.286	0.28	0.12	0.008	0.05	1.00

* Indicates that data were analyzed also by the chi-square test, XRG = X-ray grade

At 12 weeks, gross autopsy examination of two of the control rabbits showed poor healing in the form of either fragile fibrous callus or abnormal mobility with a persistent gap. There was definitely better healing in the test group which showed mature bony callus with no cartilaginous elements at 12 weeks of follow-up. Histological grading was poor in the two control rabbits sacrificed at this period but all the test rabbits showed HPG = 4 healing. Of the 14 rabbits (10 test, 4 controls) available for XRS at 12-week follow-up, all test rabbits' score was either +7 or 8 while only one control showed a score of +7. Evaluation by the chi-square test showed a *P* value of 0.05 which is statistically significant. No variation was found in both groups at 20 weeks in terms of gross autopsy, histology, and X-ray follow-up.

Histological analysis of all the rabbits in both the groups subjected to statistical analysis with the Mann-Whitney U-test including all parameters of histology showed that the average HPG in the test group was 2.82 ± 2.5 and in the control was 1.96 ± 2.36. Overall, the statistical significance was observed for vascularity (*P* value 0.031), Haversian system formation (*P* value 0.025), bone matrix formation (*P* value 0.04), cortical repair (*P* value 0.034), and the HPG (*P* value 0.037). XRS obtained at weeks 9, 10, and 12 in both the groups showed statistical significance. These findings showed that healing is better in the test group in terms of increased vascularity in the early part of healing, i.e., at approx. 2–3 weeks and in terms of bone matrix, Haversian system formation, and cortical repair in the later part of healing, i.e., at approx. 9–12 weeks between the two groups. Thus, there was overall a lower rate of healing in the control group.

## DISCUSSION

The ability of any fracture to heal is dependent on many factors. An important requirement of the fracture healing process is an adequate vascular response during which significant changes in the blood flow occur. The increase in flow occurs, either by pre-existing vasculature vasodilation or because of angiogenesis and new vessel formation. However, the endothelium acts as a semipermeable membrane in such circumstances and also represents a source of local factors that can influence local vascular tone.^6^

One such factor is nitric oxide (NO), which is derived from the basic amino acid arginine.^7^ Arginine is utilized in three pathways: NO, polyamines, and proline. All these are involved in tissue injuries and repair, inflammatory and immunological tissue injury, DNA synthesis, cell growth, and collagen production.^8^ NO is formed from arginine in the presence of enzyme nitric oxide synthase (NOS). There are three types of NOS: iNOS (inducible NOS), eNOS (endothelial NOS), and neuronal (bNOS).^9^ iNOS is inducible and calcium independent while eNOS and bNOS are calcium dependent and are constitutive. In our study, it was seen that healing in test rabbits was better at 3 weeks postoperatively mainly because of increased vascularity and better angiogenesis which had occurred due to increased NO synthesis from arginine supplementation. NO is expressed during fracture healing, and suppression of NOS impairs fracture healing.^7^ Similar results by Corbett *et al*.^6^ show that NO-mediated vasoreactivity is maximal in early phases of fracture healing, before returning to basal levels, as healing progresses. This is compatible with an initial restoration of blood flow at the fracture site by NO-dependent vasodilation of pre-existing vessels followed by growth of less NO-dependent angiogenic vessels during later stages of healing. However, vascularity was not a determinant of better fracture healing in the later stages of healing.^7^

L-arginine is metabolized to L-ornithine, which can be processed to polyamines or to proline. Polyamines are important mediators of cell growth and proline is a precursor in collagen synthesis^5^. In our study, better Haversian system and Bone matrix mineralization in the rabbits supplemented with amino acids may be attributed to increased collagen synthesis. Moreover, the oral administration of arginine in pharmacological doses also induces growth hormone (GH) and IGF-I responses.^10^ Osteoblasts have receptors for GH and these cells produce large amounts of IGF-I. IGF-I has positive effects on bone formation; firstly, it is known to stimulate the formation of osteocalcin, collagen, and noncollagenous matrix proteins by differentiated osteoblasts and secondly, it increases the number of functional osteoblasts by promoting osteoprogenitor cell replication.^11^

However, L-lysine has an equally important role to play; *in vitro* studies demonstrated that the addition of a lysine-rich 18 kDA protein to osteoblast-like cells resulted in a 1.6- to 2-fold increase in the activity of alkaline phosphatase and a slight increase in the DNA content.^11^ Clinical experiments showed that L-lysine significantly increases intestinal^12^ calcium absorption and renal conservation of absorbed calcium. Also, Schlieffer *et al*.^13^ concluded from their study on rats that NO is involved in basal calcium absorption in small intestine.

Hydroxylysine is derived from lysine and is essential for the formation of bone matrix, thus explaining better matrix formation in the test group of our study.^4^ Our results show that lysine and arginine treated rabbits have significant radiographical, morphological, and histological differences in fracture healing at 3, 8, and 12 weeks. Our results are in agreement with earlier observations of Fini *et al*.^4^ who showed similar histomorphic data that animals having fibular osteotomy and condylar femoral defects treated with lysine, arginine, and lactose have healing almost 10 days prior to those who were not supplemented. Studies by the same authors^5^ have shown that both lysine and arginine have a positive feedback on primary osteoblast culture from normal and osteopenic rats in the form of increased NO production and increased collagen production.

Our findings are in coherence with the findings of Diwan *et al*.^7^ who showed that NO is expressed during fracture healing in rats and humans as after fracture, mRNA, protein, and enzymatic activity iNOS were identified at the fracture callus with maximum activity at day 15.^9^ Thus, the initial better healing, by 3 weeks, in the test group rabbits can be explained by the fact that the iNOS activity mediates an increased vascularity at the fracture site. The mRNA activity for eNOS and bNOS was induced slightly later than that for iNOS, which was consistent with a temporal increase in the calcium-dependent NOS activity that gradually increased up to day 30. This is in accordance with our findings that all calcium-dependent processes like collagen recruitment for Haversian system formation, better bone matrix, and cortical repair were significantly better at any point of time, in the rabbits that were supplemented with arginine; however, lysine has also an important role in these processes.

The study was based on an oral administration of drugs; thus, exact bioavailabilty was lower than the dose administered. So the dose which is available at the fracture site is not known. With gene-based delivery system, viral or nonviral vectors can be used to deliver gene and gene-products/ osteoprogeniter cells at the fracture site. This needs evaluation regarding its feasibility in humans.^14^

Of course, in this preliminary phase of the study, it was not possible to separate the effect of each substance completely or to decipher the entire biological mechanisms, events, and sequences to which fracture healing is correlated.

It is certain that these results are related to other metabolic pathways of arginine or to lysine, or certain unknown factors, or to their synergistic effects. We could not obtain the statistical significance of histologic data at a particular period of study (i.e., at 4 weeks, 8 weeks, 12 weeks) separately owing to a small sample size of the subjects. But the overall analysis reveals the statistical significance which holds true according to the literature available. Crude X-ray scoring system and histological analysis scoring methods have been used in absence of a known scoring system; however, the observers were blinded to obtain the unbiased values. Yet, a significant amount of inter- and intraobserver variation cannot be ruled out. The study lacks the testing of biomechanical and immmunohistochemistry analysis, in view of lack of resources and being a preliminary pilot form of study.

Because adjuvant amino acid treatment is having inherent advantage in being nontoxic, inexpensive, and a simple oral therapy, the results obtained are inspiring. There is an area of common action between lysine and arginine for increased calcium absorption from the gastrointestinal tract and effective participation in collagen synthesis which is incriminated in rapid fracture healing along with calcium-dependent activity of certain isoenzymes like NOS. Potential areas of application of this form of therapy are during biological failure of unions, nonunions, chronic ischemic conditions like diabetes,^15^ atherosclerosis, vasculitis, etc. and increasing calcium absorption in situations like osteoporosis.
